# Mg^2+^ Doping Effects on the Structural and Dielectric Properties of CaCu_3_Ti_4_O_12_ Ceramics Obtained by Mechanochemical Synthesis

**DOI:** 10.3390/ma14051187

**Published:** 2021-03-03

**Authors:** Piotr Dulian, Wojciech Bąk, Mateusz Piz, Barbara Garbarz-Glos, Olena V. Sachuk, Krystyna Wieczorek-Ciurowa, Agata Lisińska-Czekaj, Dionizy Czekaj

**Affiliations:** 1 Faculty of Chemical Engineering and Technology, Cracow University of Technology, 24 Warszawska St., 31-155 Cracow, Poland; kwc@pk.edu.pl; 2 Institute of Technology, Pedagogical University of Cracow, 2 Podchorążych St., 30-084 Kraków, Poland; wojciech.bak@up.krakow.pl (W.B.); barbara.garbarz-glos@up.krakow.pl (B.G.-G.); 3 Department of Inorganic and Analytical Chemistry, Faculty of Chemical Technology and Engineering, West Pomeranian University of Technology, al. Piastów 42, 71-065 Szczecin, Poland; mateusz.piz@zut.edu.pl; 4 Institute of Technology, The Jan Grodek State University in Sanok, 6 Reymonta St., 38-500 Sanok, Poland; 5 National Academy of Sciences of Ukraine, Institute for Sorption and Problems of Endoecology, 13 General Naumova St., 03164 Kyiv, Ukraine; slena951@ukr.net; 6 Faculty of Mechanical Engineering and Ship Technology, Gdańsk University of Technology, 11/12 Narutowicza St., 80-233 Gdańsk, Poland; agata.czekaj@pg.edu.pl (A.L.-C.); dioczeka@pg.edu.pl (D.C.)

**Keywords:** mechanochemical synthesis, ceramics, CaCu_3_Ti_4_O_12_ (CCTO), dielectric spectroscopy, relaxation processes

## Abstract

In this study, ceramic CaCu_3_Ti_4_O_12_ (CCTO) and CaCu_3−x_Mg_x_Ti_4_O_12_ solid solutions in which 0.1 ≤ x ≤ 0.5 were prepared by the mechanochemical method, realized by a high-energy ball milling technique. The effects of the Mg^2+^ ion concentration and sintering time on the dielectric response in the prepared ceramics were investigated and discussed. It was demonstrated that, by the use of a sufficiently high energy of mechanochemical treatment, it is possible to produce a crystalline product after only 2 h of milling the mixture of the oxide substrates. Both the addition of magnesium ions and the longer sintering time of the mechanochemically-produced ceramics cause excessive grain growth and significantly affect the dielectric properties of the materials. The X-ray diffraction (XRD) analysis showed that all of the as-prepared solid solutions, CaCu_3−x_Mg_x_Ti_4_O_12_ (0.0 ≤ x ≤ 0.5), regardless of the sintering time, exhibit a cubic perovskite single phase. The dielectric study showed two major contributions associated with the grains and the grain boundaries. The analysis of the electric modules of these ceramics confirmed the occurrence of Maxwell–Wagner type relaxation, which is dependent on the temperature.

## 1. Introduction

Due to the ongoing technological trends of miniaturization, the demand for temperature-stable high permittivity capacitor materials has increased [1]. Most of the currently available capacitor materials which exhibit a high real part of dielectric permittivity (ε’) are based on ferroelectrics, such as BaTiO_3_, or relaxor ferroelectrics including, e.g., Pb(Mg_1/3_Nb_2/3_)O_3_, Pb(Zn_1/3_Nb_2/3_)O_3_, and Pb_1−x_La_x_(Zr_1−y_Ti_y_)O_3_ [2]. However, these kinds of materials exhibit a strong temperature dependence of ε’, limiting their straightforward application in electronic devices. Therefore, the high dielectric permittivity of materials has received increased interest for many applications in recent years. 

CaCu_3_Ti_4_O_12_ (CCTO) is one of the most interesting materials with high ε’, and has been intensively investigated due to its potential technological applications, and for academic reasons [3]. Complex cubic perovskite-like oxide CCTO exhibits extraordinarily high permittivity (ε’~10^4^–10^5^), which is temperature-independent (over −100–250 °C) and frequency-independent (typically from 0.01Hz to 10^7^ Hz range), without any structural phase transition [4,5]. Unfortunately, the dielectric loss (tan δ) of CCTO is still much higher than the standard acceptable value for practical applications, and it strongly depends on various factors, such as the preparation method, sintering temperature and time, sintering atmosphere, doping ions, and cation stoichiometry. An additional difficulty is the fact that the mechanism of the unique dielectric properties of this compound is still clearly unexplained. Many models have been proposed to explain the origin of the giant dielectric permittivity, such as the Maxwell–Wagner theory or the Internal Barrier Layer Capacitor (IBLC) model [6,7,8]. Intensive investigations tend to use the IBLC model to explain the origin of the dielectric properties, which suggests that the high value of dielectric permittivity is associated with semiconducting grains and insulating grain boundaries. Therefore, it seems that the change of electrical properties of grains and/or at grain boundaries will affect the dielectric behaviour of CCTO considerably. Several methods have been proposed to reduce the dielectric loss, such as the alteration of the processing conditions [9], doping processes [10,11], and an addition of insulating oxides, such as ZrO_2_ [12], SiO_2_ [13], TiO_2_ [14]. However, the results are mostly not desirable, i.e., when the dielectric permittivity of CCTO after modification was increased, the value of dielectric losses also increased, rather than being reduced. In addition, it was noted that the electrical properties of modified and pure CCTO depend strongly on the processing routes, due to the different morphology and microstructure of the produced electro-ceramics [15,16,17]. 

Recently, the use of magnesium (Mg/MgO) as a CCTO modifier has been reported by some groups, and it seems to be an effective way to improve its dielectric properties. The substitution of Mg^2+^ ions at the Cu^2+^ site of the CCTO crystal lattice is one of the most effective methods to reduce its dielectric losses without reducing the dielectric permittivity at the same time. It was found that the permittivity is enhanced from 14,300 to above 16,000 by doping Mg^2+^ ions at the Cu^2+^ site at room temperature, and at a frequency of 1 kHz. Notably, the tan δ is reduced from 0.2 for the pure sample of the CCTO to below 0.071 for the doped samples [18]. Rahman et al. [19,20,21] also indicates that Mg^2+^ ion doping at different cation sites in the crystal structure of the CCTO can alter and improve the properties of this material. An increase in the dielectric permittivity and a large decrease in the dielectric losses were simultaneously achieved in the Ca_1−x_Mg_x_Cu_3_Ti_4_O_12_ ceramics with x = 0.05 within the frequency range between 1 kHz and 20 kHz by Li et al. [22]. Furthermore, MgO has been identified as a good sintering aid, promoting densification during the sintering stage. The addition of this oxide significantly changes the microstructure of the produced powder, which has a positive effect on its dielectric properties [23,24,25]. 

An important difficulty in the Mg^2+^ doping of CCTO is its low synthesis temperature (about 1100 °C) compared to similar compounds with a perovskite structure, like CaTiO_3_ or SrTiO_3_, which causes the low solubility of modifier ions in the CCTO crystal lattice. The use of a higher temperature on the one hand solves this problem, but on the other, a number of additional complications arise, e.g., a lack of stoichiometry, a change in the copper oxidation state, structure defects, excessive grain growth, or the appearance of additional phases, e.g., Cu_2_O, CuO. These factors negatively affect the dielectric properties of these electro-ceramics, but they can be limited by the use of other/wet synthesis methods, e.g., sol-gel [26,27]. The inherent problems of wet techniques are, among others, high nuisance for the natural environment due to the used types of cation precursors and solvents, the large amount of waste, and the different solubility of the cation precursors in the same solvent, which limits the precise regulation of doping. New strategies for the fast and efficient synthesis and doping of perovskite compounds remain a challenge. In this study, we use a solvent-free mechanochemical approach to synthesize Mg^2+^ ion-doped CaCu_3_Ti_4_O_12_. Compared to the previously-presented methods, mechanochemistry seems to be an interesting approach to the synthesis of this type of ceramic materials [28,29]. A characteristic feature of this method is the fact that high-energy milling is used to initiate the chemical reactions. During such a treatment, the reactivity of solids increases significantly due to the presence of various types of structural defects, interphases, and relaxation phenomena, enabling processes to take place under non-equilibrium conditions, thereby performing the simple, dry, time-convenient, one-step solid-state synthesis of various types of compounds. In addition, mechanochemical processes stimulate diffusion in the solid, which allows us to avoid solubility problems in solid solutions. 

Our work demonstrated that Mg^2+^ ion-doped CCTO perovskite can be successfully synthesized by a mechanochemical approach in a short time by a simple milling process. The influences of different doping ratios and sintering conditions on the microstructure and dielectric properties were analysed and discussed. Our effort was focused on finding an effective strategy to enable the simplification and reduction of the sample preparation time, in order to improve the functional properties of the CCTO electro-ceramics by introducing a modifier ion into its crystal lattice.

## 2. Materials and Methods

### 2.1. Mechanochemical Synthesis

The ceramic samples of CaCu_3_Ti_4_O_12_ (CCTO) and the solid solutions Ca(Cu_3−x_Mg_x_)Ti_4_O_12_, where 0.1 ≤ x ≤ 0.5, were synthesized by the solid-state mechanochemical method realized by high-energy ball milling. Calcium oxide (Sigma Aldrich, 99.9%, Poznan, Poland), TiO_2_ (Sigma Aldrich, 99.7%), CuO (Fluka, 98%, Charlotte, NC, USA) and MgO (Chempur, 99.7%, Piekary Śląskie, Poland) were used as the primary raw materials. All of the oxides were first heat treated in order to remove hydroscopic water. These precursors of stoichiometric quantity were hand-mixed in an agate mortar for thirty minutes in order to obtain a homogenous mixture, and were then subjected to high-energy milling in a Fritsch GmbH. *Pulverisette-6* planetary ball mill. The mechanochemical treatment of the powders was carried out in air, with the vessel and balls (Ø = 10 mm) being made of ZrO_2_. The rotation speed was 550 rpm, and the grinding time was 2 h, with a balls-to-powder weight ratio (BPR) of 40:1. The as-prepared ceramic powders were pressed into pellet discs (8 mm in diameter, 2 mm in thickness) and sintered in an air atmosphere at 1075 °C for 2 h and 10 h, respectively. 

### 2.2. Characteristics of the Materials

A variety of techniques were used to characterize the prepared materials. The structure of the perovskite materials was determined by X-ray powder diffraction (XRD) measurements performed with an Empyrean II (Malvern PANalytical, Almelo, the Netherlands) diffractometer, using a CuKα lamp (λ = 1.54178 Å) and a graphite monochromator. The measuring range was between 10° < 2 theta <90°, with a step-width of 0.01°. The qualitative identification of the various phases was performed based on the conformity of the data obtained with those in the PDF4+ cards. The crystal unit cell parameters of the selected solid solutions were indexed using the POWDER program; the XRD data were refined by the Refinement program of the DHN/PDS package software.

A high-resolution scanning electron microscope (Hitachi S4700, Tokyo, Japan) with an EDS (Energy-Dispersive X-Ray Spectroscopy) was used to determine the morphology and homogeneity of the synthesized ceramic materials.

The complex dielectric permittivites and electrical modulus were measured using a Novocontrol Alpha High Resolution Dielectric Analyzer (Montabaur, Germany) in the temperature range from −100 °C to 200 °C, in which nitrogen was used as a heating and cooling agent. The frequencies varied from 0.1 Hz to 10 MHz. Silver paint was used on the polished surfaces as electrodes. 

## 3. Results and Discussion

### 3.1. Mechanochemical Synthesis of Ceramics Powders

**Figure 1:**
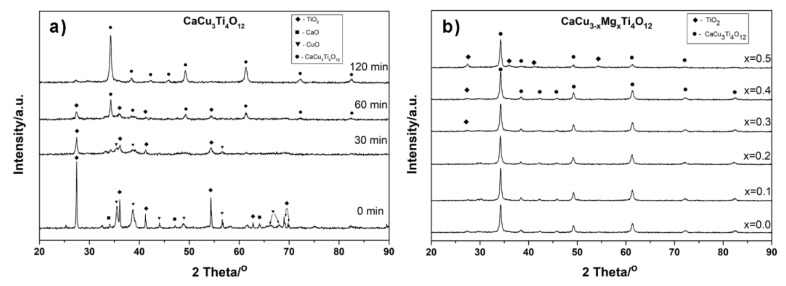
XRD diffraction patterns of: (**a**) substrate powders after different times of mechanochemical processing; (**b**) CaCu_3−x_Mg_x_Ti_4_O_12_ solid solutions with 0.1 ≤ x ≤ 0.5, prepared by the mechanochemical synthesis method.

Figure 1 illustrates the synthesis of the CaCu_3_Ti_4_O_12_ perovskite compound and CaCu_3−x_Mg_x_Ti_4_O_12_ solid solutions by the mechanochemical method. The X-ray diffraction (XRD) patterns of the substrate powders after different times of high-energy ball milling are shown in Figure 1a. 

**Figure 2:**
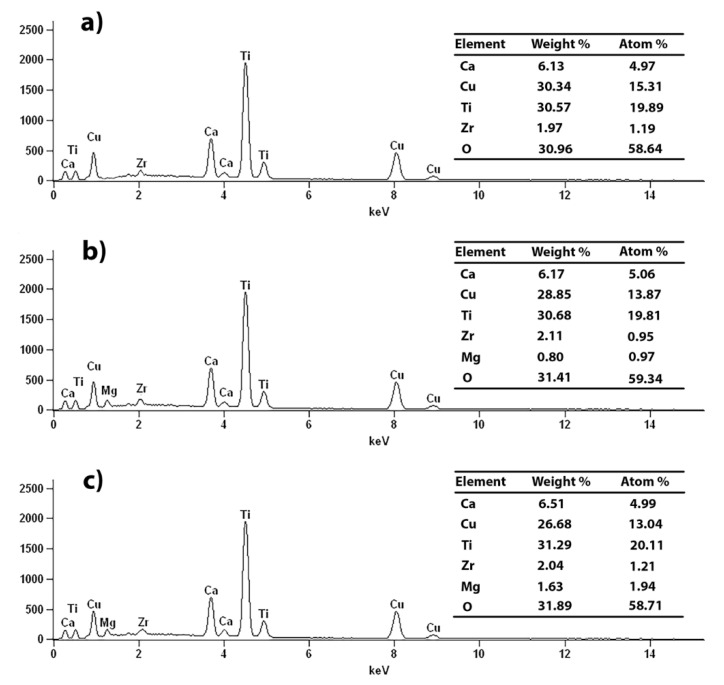
EDS analysis of the mechanochemically-prepared samples: (**a**) CaCu_3_Ti_4_O_12_; (**b**) CaCu_2.8_Mg_0.2_Ti_4_O_12_; (**c**) CaCu_2.6_Mg_0.4_Ti_4_O_12_.

The gradual disappearance of the X-ray diffraction reflections corresponding from the substrates during the mechanochemical treatment, and the appearance of the reflections that indicate the formation of a crystalline product—CCTO—are clearly visible. After two hours of the high-energy milling of the powder, only the perovskite phase is noticeable. The synthesis of CaCu_3−x_Mg_x_Ti_4_O_12_ solid solutions with different modifier concentrations of 0.1 ≤ x ≤ 0.5 was performed in an analogous manner. The XRD patterns of the Mg^2+^ ion-doped CCTO ceramics are shown in Figure 1b. They confirm the presence of a primary CaCu_3_Ti_4_O_12_ phase (JCPDS 75-2188) in all of the prepared ceramics. A small amount of a secondary TiO_2_ phase was observed in the samples in which the concentration of magnesium oxide was high. Singh et al. [30] previously reported the benefits of using mechanochemical synthesis to produce magnesium-doped CaCu_3_Ti_4_O_12_. It has been shown that the grinding of the substrates in ethanol led to a decrease in the synthesis temperature of the compound (800 °C), and positively affected the dielectric properties of the ceramics. In this study, a significantly higher milling energy was used, which led to the formation of the crystalline product only due to the mechanochemical treatment of solids. The downside of using such high-energy milling is the presence of impurities in the product from abrasive milling equipment components. Hence, milling balls and a vessel made of ZrO_2_ were used, which has a minor influence on the dielectric properties of the ceramic [29]. In this case, no X-ray diffraction reflections from zirconia were observed on the diffraction patterns (Figure 1), which indicates a low amount of this pollution, i.e., below the detection limit of the XRD method. The elemental analysis of the samples performed by EDS showed the presence of zirconium at around 2 wt %. Figure 2 presents the results of the elemental analysis studies for the selected samples, i.e., the pure CaCu_3_Ti_4_O_12_ compound and two solid solutions: CaCu_2.8_Mg_0.2_Ti_4_O_12_ and CaCu_2.6_Mg_0.4_Ti_4_O_12_. 

All of the EDS peaks from individual elements (Ca, Cu, Mg, Ti, O) and from the above-mentioned zirconia impurity were detected. The percentage of various elements in the tested samples was determined from the whole surface of the samples. The values of the individual elements for each sample, presented in the tables (Figure 2), are consistent with the expected stoichiometry of compounds. 

**Figure 3:**
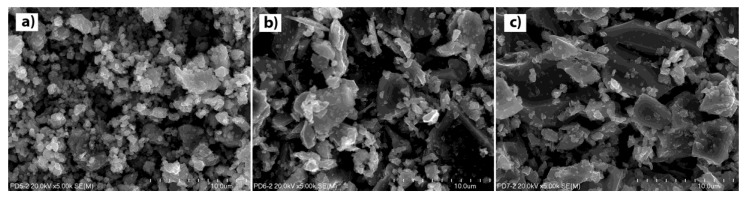
Scanning electron microscopy photographs of the surface of the CaCu_3−x_Mg_x_Ti_4_O_12_ samples sintered at 1075 °C for 2 h, where: (**a**) x = 0.0; (**b**) x = 0.2; (**c**) x = 0.4.

The ceramic samples, after the mechanochemical treatment, were subjected to sintering at 1075 °C for different lengths of time—2 and 10 h—in order to increase the density of the ceramics. The length of calcination time significantly affected the morphology of the ceramic powders, in particular the size of the crystallites. The analysis of the chemical composition of the tested materials before and after the high-temperature treatment process showed no changes in the content of individual elements. None of the tested samples showed any deviations from the stoichiometric composition, regardless of the sintering time (2 or 10 h). The surface morphologies of the selected samples—CaCu_3_Ti_4_O_12_, CaCu_2.8_Mg_0.2_Ti_4_O_12_ and CaCu_2.6_Mg_0.4_Ti_4_O_12_, sintered at 1075 °C for 2 h—are shown in Figure 3.

**Figure 4:**
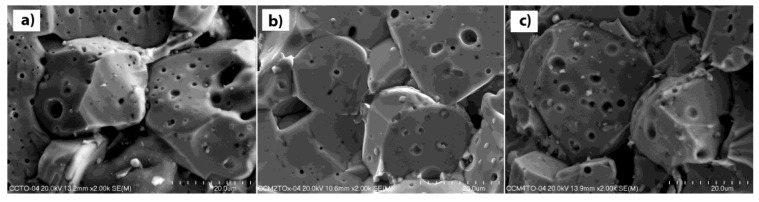
SEM photographs of the surface of the CaCu_3−x_Mg_x_Ti_4_O_12_ samples sintered at 1075 °C for 10 h where (**a**) x = 0.0; (**b**) x = 0.2; (**c**) x = 0.4.

The effect of the addition of magnesium ions on the shape and size of the crystallites is clearly visible; the higher concentration of the modifier and the larger grains with irregular shapes are observed. Mechanochemically-prepared CCTO powder is characterized by fine-grained microstructure with the smallest particle size, good homogeneity, and similar shape. The average crystallite size is around 150–200 nm. The ceramics containing the modifier are characterized by a different surface morphology; they consist of two types of crystallites: large irregular grains and very small grains with various shapes and sizes. The presence of the Mg^2+^ dopant seems to promote grain growth. The size of the crystallites varies from 2 µm for the sample of CaCu_2.9_Mg_0.1_Ti_4_O_12_ to 10 µm for the sample of CaCu_2.5_Mg_0.5_Ti_4_O_12_ (not shown). Additionally, the higher concentration of the modifier reduces the proportion of small grains in the material. Extending the heat treatment time significantly effects on the morphology of the ceramics. Figure 4 shows the SEM image for the surface morphologies of the undoped CCTO sample and the magnesium-doped ceramics sintered at 1075 °C for 10 h. The differences in comparison with the corresponding materials calcined for 2 h are clearly visible. The microstructure of all of the investigated ceramics consisted of very large grains in the size range of 20–40 µm, which is a consequence of long-term high-temperature treatment. Additionally, a large number of sinters and pores are observed in both the undoped and doped samples. The similar observations in the microstructure of such ceramics have already been reported by previous researchers for samples prepared by the conventional high-temperature synthesis method [22,23]. The mechanism for the grain growth of CCTO in high-temperatures is complex, and is based on the CuO liquid sintering phase. The Cu-rich phases caused from the deoxidization of the liquid Cu_2_O, which is initially reduced from CuO at a temperature of around 900 °C. These phases segregate at the grooved grain boundaries, and lead to the abnormal grain grow and porous structure. Additionally, in samples containing the modifier in the form of magnesium ions, CuO-MgO solid solutions may form. Such a solid solution with a higher melting temperature can limit the mobility of the ions (diffusion) in the solid phase, and can distort the crystal lattice of the matrix. 

**Figure 5:**
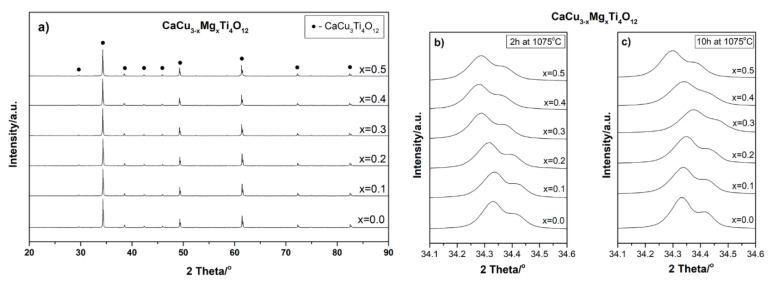
XRD diffraction patterns of (**a**) the mechanochemically-prepared CaCu_3−x_Mg_x_Ti_4_O_12_ solid solutions with 0.1 ≤ x ≤ 0.5, subjected to subsequent heat treatment for 2 h at 1075 °C; zoom of the 220 peak for materials subjected to subsequent heat treatment for: (**b**) 2 h at 1075 °C and (**c**) 10 h at 1075 °C.

Figure 5a shows the XRD patterns of mechanochemically-prepared CaCu_3−x_Mg_x_Ti_4_O_12_ powders with different Mg^2+^ ions concentrations subjected to subsequent sintering at 1075 °C for 2h. The analogous samples, sintered at the same temperature for 10 h, are not shown in the paper due to their high similarity. In all of the diffraction patterns, regardless of the amount of modifier and sintering time, only one CaCu_3_Ti_4_O_12_ phase is visible (JCPDS 75-2188). Comparing these results to the diffraction patterns of the powders after mechanochemical treatment (Figure 1), the differences are clearly visible. The subsequent thermal treatment of the powders led to the reaction of the residual substrates; there are no visible diffraction reflections corresponding to the secondary phases (e.g., TiO_2_) or impurities from the grinding media (ZrO_2_). Additionally, the degree of the crystallinity of the ceramics has improved, as evidenced by the lower half-widths and higher intensities of the CCTO diffraction reflections. 

**Figure 6:**
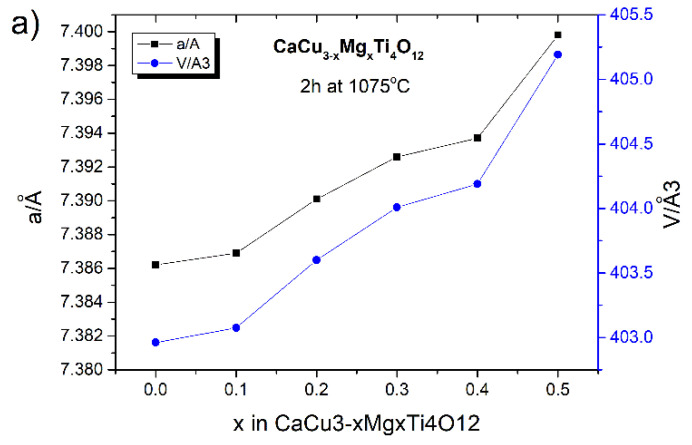

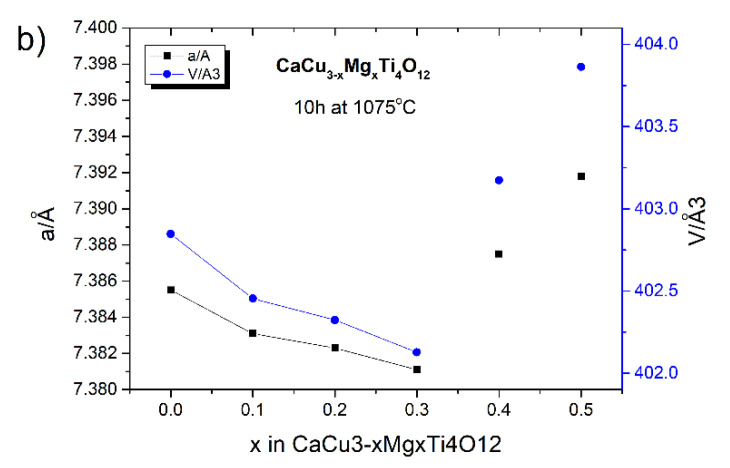
Lattice parameters of the mechanochemically-prepared CaCu_3_Ti_4_O_12_ and CaCu_3−x_Mg_x_Ti_4_O_12_ (0.1 ≤ x ≤ 0.5) samples, as determined from the XRD data, subjected to subsequent heat treatment for different times: (**a**) 2 h at 1075 °C; (**b**) 10 h at 1075 °C.

The diffraction peaks in the XRD patterns for the CaCu_3_Ti_4_O_12_ and all of the CaCu_3−x_Mg_x_Ti_4_O_12_ solid solution (0.1 ≤ x ≤ 0.5) powders, regardless of the sintering time, are perfectly indexed to the bcc structure with the space group *Im3*. The lattice parameter values of all of the investigated samples were calculated, and are summarized in Figure 6. A detailed analysis of the diffraction data showed slight differences in the values of the CCTO unit cell parameters due to the doping and the length of the sintering time. 

In the case of the mechanochemically-produced solid solutions sintered for 2 h at 1075 °C, the value of the unit cell parameter (a) increases with the amount of modifier added. The calculated lattice parameters of these samples were 7.3862(4), 7.3869(5), 7.3901(3), 7.3926(1), 7.3937(2), and 7.3998(6) Å for the samples with x = 0.0, 0.1, 0.2, 0.3, 0.4, and 0.5, respectively. The unit cell volumes [Å^3^] for these samples are as follows: 402.9612; 403.0757; 403.5998; 404.0095; 404.1899; and 405.1911. This is also visible in a slight shift of the CCTO diffraction reflections towards lower values of two Theta angles (Figure 5b). Despite the similar ion radii in the same coordination, the partial substitution of magnesium ions (Mg^2+^ = 0.57 Å) for copper ions (Cu^2+^ = 0.57 Å) in the CCTO crystal lattice causes a slight change of unit cell parameter (a) because of the increase in the surface energy of the crystal. This is attributed to the chemical bond strength, defined as the ratio of the valence over the internuclear distance; the chemical bond of Mg–O (1.09) is larger than that of Cu–O (1.01). The obtained results are in accordance with the results reported by other authors [24]. Extending the time of the high-temperature treatment to 10 h also changes lattice parameter (a) of the CCTO unit cell. In this case, for smaller amounts of the modifier (x ≤ 0.3), this value decreases, while for larger amounts of Mg^2+^ (x ≥ 0.4) the (a) value increases (see Figure 5c and Figure 6. The sequence of lattice parameters of these samples was calculated: 7.3855(4), 7.3831(7), 7.3823(8), 7.3811(1), 7.3875(3), and 7.3898(7) Å. The unit cell volumes [Å^3^] for these samples are as follows: 402.8466, 402.4540, 402.3232, 402.1270, 403.1740, and 404.0570. The significant differences of the lattice parameters were observed. These results, in comparison with the previous ones, indicate that—in this case—other substitutional types of solid solutions may be formed. In addition, the long-term heat treatment of ceramics at a high temperature in an atmosphere containing oxygen (air) may also damage the crystal structure through the formation of various types of structural defects, including oxygen vacancies, etc. [27]. Moreover, the long-term high-temperature treatment of the ceramics could stimulate diffusion processes in the solid, and could thus lead to the substitution of larger zirconium ions (Zr^4+^ = 0.59 Å), from the grinding media, into the CCTO crystal lattice. 

### 3.2. Dielectric Properties

**Figure 7:**
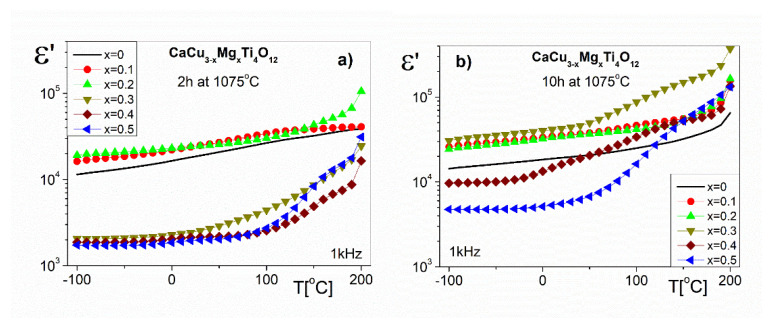
The temperature dependence of the real part of the dielectric permittivity for CaCu_3−x_Mg_x_Ti_4_O_12_ sintered at 1075 °C for 2 h (**a**) and for 10 h (**b**).

The temperature dependences of the real part of the dielectric permittivity (ε’) for the chosen frequency (1 kHz) are shown in Figure 7a,b for the solid solutions sintered at 1075 °C for 2 h and 10 h, respectively. The values of the function ε’(T) slightly increase below room temperature (RT) for all of the investigated samples. Below room temperature (RT), the values of the ε’(T) function do not change significantly for all of the investigated samples. The ε’ values at RT in Figure 7a for the CaCu_3−x_Mg_x_Ti_4_O_12_ ceramics with the lower Mg^2+^ ions content (x ≤ 0.2) are approximately 18,000 and 24,000 for x = 0 and x = 0.2, respectively. One can see that the ε’ for the samples with higher amounts of Mg^2+^ ions (x ≥ 0.3) shows ten-times lower values (~2000). In the temperature range above RT, the ε’ values for CaCu_3−x_Mg_x_Ti_4_O_12_ ceramics with x ≥ 0.3 increase rapidly. The high temperature treatment (10 h at 1075 °C) of the samples increases the values of the ε’ (Figure 7b). For the chosen ceramics at RT, the ε’ values are approximately 19,000, 34,000, 42,000 and 5600 for x = 0, 0.2, 0.3 and 0.5, respectively. These results are usually observed in the literature [8,9,26,30]. The high ε’ values may be interpreted as an internal barrier layer capacitor (IBLC) effect [31]. According to this model, CaCu_3−x_Mg_x_Ti_4_O_12_ ceramics are constituted of semiconducting grains and insulating grain boundary layers. The studies of the ceramics using impedance spectroscopy allowed us to separate the grain and the grain boundary contributions [26]. All of the parameters of the equivalent circuit model show a variation in the resistance of the grain and the grain boundary with the amount of MgO used during the ceramic synthesis. According to the literature [32] data, it can be concluded that the grain boundary resistance of the investigated ceramics is closely related to the concentration of oxygen vacancies at the grain boundaries, the charge of which contributes to the polarization mechanism. The value of this resistance depends on the thermal treatment (the sintering time and sintering temperature) of the CaCu_3−x_Mg_x_Ti_4_O_12_ ceramics. As the content of Mg^2+^ ions in the samples increases, the grain boundary resistance increases. This is due to the ability of the Mg^2+^ doping ions to inhibit oxygen loss in the samples. Therefore, the increase of ε’ with Mg^2+^ ion substitution may be attributed to the formation of the insulating grain boundary layers with an Mg-rich phase. The results reported in the literature [8,15,26,27,30] show that the high ε’ values of CCTO ceramics are caused by the electrical response of the grain boundaries. In all probability, higher amounts of Mg^2+^ ions in CaCu_3−x_Mg_x_Ti_4_O_12_ ceramics cause an increase in the grain boundary capacitance, which leads to an increase in the values of ε’. On the other hand, according to the results of the microstructure analysis (Section 3.1), it was found that the ε’ values depend on the grain size, their shape, and their number of pores. Thus, the properties of the microstructure (e.g., the number of pores) may result in decreased ε’ values. The highest values of ε’ in the whole temperature range were observed for CaCu_3−x_Mg_x_Ti_4_O_12_ ceramics with an Mg^2+^ ion content of x = 0.3, sintered at 1075 °C for 10 h (Figure 7b). 

**Figure 8:**
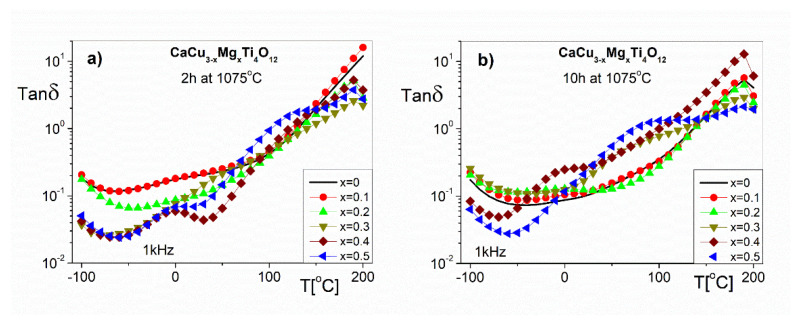
The temperature dependence of the loss angle tangent for CaCu_3−x_Mg_x_Ti_4_O_12_ sintered at 1075 °C for (**a**) 2 h and (**b**) for 10 h.

Complementary to the polarization phenomenon represented by the ε’ are the dielectric losses expressed by the imaginary part of the dielectric permittivity or Tanδ. The temperature dependences of the Tanδ at 1 kHz are shown in Figure 8. These dependencies describe the dielectric properties related to the energy loss processes of the electric field in the investigation samples. The values of Tanδ at RT (Figure 8a) for CaCu_3−x_Mg_x_Ti_4_O_12_ ceramics in the entirety of the doping concentration range (x = 0–0.5) are approximately (0.2–0.05), respectively. As can be seen in Figure 8b, the Tanδ values of the ceramics sintered at 1075 °C for 10 h are larger than 0.1, which excludes them in applications as capacitors [1]. The Tan δ (T) functions for all of the investigated samples in the temperature range below RT show a local minimum caused by the existence of dielectric relaxation phenomena (in the range of the higher frequencies). We can also see that, at temperatures higher than RT, the Tan δ increases monotonically to 190 °C, which is caused by the increase in the dc conductivity in the bulk samples, and low frequency relaxation behavior. 

**Figure 9:**
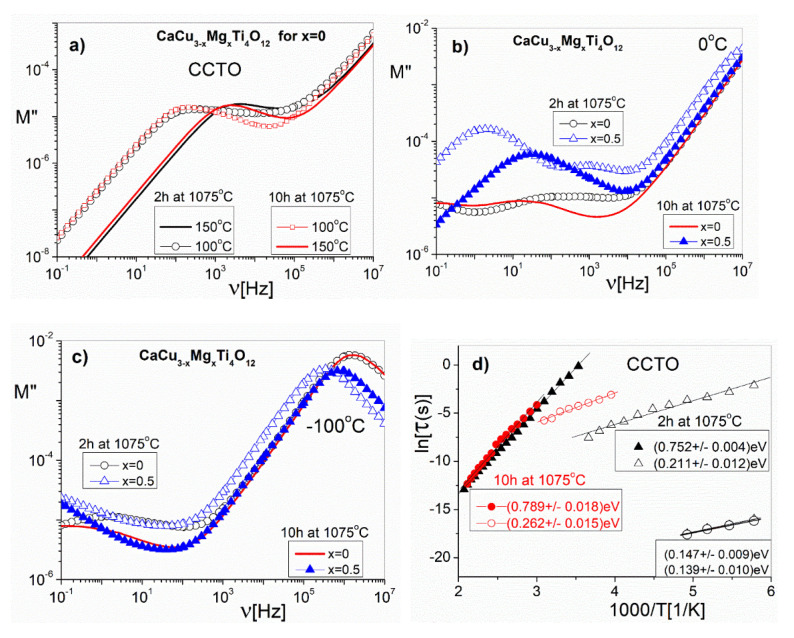
Frequency dependence of the imaginary part of the electric modulus (M’) of the CCTO (sintered at 1075 °C for 2 h and 10 h) samples at two chosen temperatures (**a**); CaCu_3−x_Mg_x_Ti_4_O_12_ (for x = 0 and x = 0.5) at a 0 °C temperature (**b**); CaCu_3−x_Mg_x_Ti_4_O_12_ (for x = 0 and x = 0.5) at a −100 °C temperature (**c**); and the temperature dependence of the relaxation times obtained from the frequency-dependent plots of M” (**d**).

In order to explore the Mg^2+^ ion doping effects on the dielectric properties of the grains and the grain boundaries, we used the complex electric modulus formalism (M* = M’ + iM”). The M* formalism describes the phenomena of electric charge transport and dielectric relaxation in these types of materials [32]. The electric modulus studies in the frequency domain correspond to the relaxation of the electric field in the material when the electric displacement remains constant [33]. The formalism of the electrical modulus is very sensitive to small changes in the value of the local polarization (capacitance), so it is—among others—the most suitable for the description of the electrical properties of grains and grain boundaries in ceramic (or inhomogeneous) materials. The frequency dependences of the M” (in a log–log scale) are shown in three separate figures, in order to represent the different temperature ranges and different samples. Figure 9a shows the M”(ν) functions at the chosen temperatures of 100 °C and 150 °C for the CCTO samples with different sintering times. Both samples show the maximum, which shifts systematically in the temperature range from RT to 200°C, with increasing frequency. The shift of the M” maximum corresponds to the so-called ‘conductivity relaxation process’ [34]. We attribute the conduction process to the electrical properties of grain boundaries. In the temperature range from −100 °C to 50 °C can be seen another low-frequency dielectric relaxation process (Figure 9b). In the case of the CCTO sample, the changes in the maximum value of M” are slight, which corresponds to relatively large values of the sample capacitance. On the other hand, CaCu_3−x_Mg_x_Ti_4_O_12_ ceramics with an Mg^2+^ ion content of x = 0.5 have lower capacitance values compared to CCTO, which results in lowered ε’ values. These low-frequency relaxation processes may be related to the movement of free charges through the sample towards the opposite electrode in the presence of an external ac electric field. This creates a macrodipole of which the oscillations give rise to relaxation and conduction processes [35,36]. We attribute this process to Maxwell–Wagner relaxation because the samples consist of heterogeneous dielectric components (grains and grain boundaries) that have different conductivities. Moreover, we notice that the conductivity relaxation process (maximum M”) in CaCu_3−x_Mg_x_Ti_4_O_12_ solid solutions with higher amounts of Mg^2+^ ions (x ≥ 0.3) appears at a lower temperature than that of CCTO (Figure 9b). This means that Mg^2+^ ion doping decreases the activation energy of the conduction process. Figure 9c show an example the frequency dependences of the M” with a temperature range of −100 °C to −70 °C for CaCu_3−x_Mg_x_Ti_4_O_12_ ceramics with an Mg^2+^ ion content of x = 0 and x = 0.5. The values of M” at the maximum confirm the presence of a small capacitance, which is responsible for the dielectric properties of the grains. These maxima, in the case of the grains, are about 100 times greater than those corresponding to the grain boundaries. This indicates that the grain capacitance is approximately 100 times smaller than that of grain boundaries. 

This maximum (Figure 9a–c) indicates a transition from the short range to long range mobility of the charge carriers, with decreasing frequency. The characteristic relaxation time, τ = (2πν_m_)^–1^, is the most probable conductivity relaxation time determined from the maximum frequency (ν_m_) of the M”(ν) functions. The relaxation time, τ, exhibits a thermally-activated dependence. This generally follows the Arrhenius law: τ = τ_o_exp(E_a_/k_B_T), where τ_o_ is the pre-exponential factor and E_a_ denotes the activation energy for dielectric relaxation. In Figure 9d, we show the temperature dependence of the relaxation times from the M”(ν) spectra for the CCTO (sintered at 1075 °C for 2 h and 10 h) samples. Based on the Arrhenius plot, it can be determined that the grain response maximum M”(ν) is observed at a low temperature and high frequency range (above 1 MHz), whereas for the grain-boundary, these responses of M”(ν) correspond to higher temperatures (above RT) and a low frequency range (below 100 kHz). 

**Figure 10:**
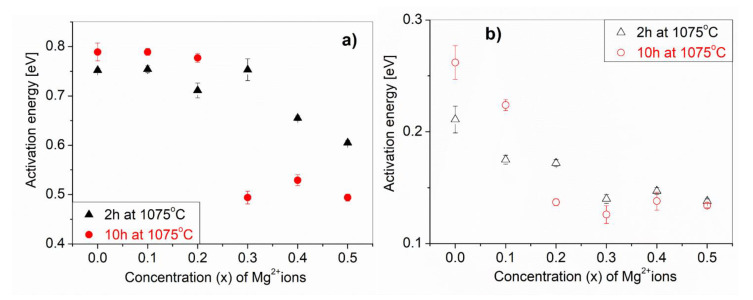
The dependence of the activation energy of the conduction process (**a**) and the dielectric relaxation (**b**) from the amounts of Mg^2+^ (x) for CaCu_3−x_Mg_x_Ti_4_O_12_ solid solutions.

Figure 10 shows the dependence of the activation energy of the conduction process (a) and dielectric relaxation (b) from the amounts of Mg^2+^ ions (x) for CaCu_3−x_Mg_x_Ti_4_O_12_ solid solutions. We can clearly see that Mg^2+^ ion doping decreases the activation energy of both processes. The activation energy of the conductivity relaxation process corresponding to the grain boundaries for the CCTO sample was found to be approximately 0.8eV. Sinclair et al. [31] determined an activation energy of the CCTO grain boundaries equal to 0.60 eV. For the CaCu_3−x_Mg_x_Ti_4_O_12_ solid solutions with higher amounts of Mg^2+^ ions (x ≥ 0.3) and a high temperature treatment (10 h at 1075 °C), this activation energy decreased to about 0.5eV (Figure 10a). The grain activation energy 0.140eV of our CCTO sample is different from the 0.08eV of the CCTO grains [32]. From the slopes of the fitted straight lines, we obtain the activation energies (Figure 10b) for the Maxwell–Wagner relaxation, which generally refers to interfacial polarization occurring in electrically-inhomogeneous systems. 

## 4. Conclusions

In conclusion, a novel route for the improvement of the dielectric properties of CaCu_3_Ti_4_O_12_ ceramics was proposed by doping with Mg^2+^ and using a mechanochemical synthesis method. It was demonstrated that crystalline CaCu_3_Ti_4_O_12_ and CaCu_3−x_Mg_x_Ti_4_O_12_ (0.1 ≤ x ≤ 0.5) perovskites can be successfully synthesized by the mechanochemical approach in a short time (2 h), by a simple milling process. The influence of the sintering time (2 and 10 h at 1075 °C) on the microstructure and dielectric properties of the ceramics was also determined. Both the addition of magnesium ions and the longer sintering time of the mechanochemically-produced ceramics cause excessive grain growth and significantly affect the dielectric properties of the materials. The results show that all of the as-prepared solid solutions of CaCu_3−x_Mg_x_Ti_4_O_12_ (0.0 ≤ x ≤ 0.5), regardless of the sintering time, exhibit a cubic perovskite single phase. We found that the samples are electrically inhomogeneous because two conduction processes in the electrical modulus spectra were detected. We attributed them to grain and grain boundary effects, and calculated their activation energies from the Arrhenius law. We also observed the presence of Maxwell–Wagner type relaxation. 

## Data Availability

The data presented in this study are available on request from the corresponding author.
